# The Impact of Changes in Direct Care Staffing Policies and Outcomes for Assisted Living Residents With Dementia

**DOI:** 10.1093/geroni/igaa057.2527

**Published:** 2020-12-16

**Authors:** Kali Thomas, Portia Cornell, Wenhan Zhang, Paula Carder, Lindsey Smith, Cassandra Hua, Momotazur Rahman

**Affiliations:** 1 Brown University, Providence, Rhode Island, United States; 2 Providence VA Medical Center, Providence, Rhode Island, United States; 3 Portland State University, Portland, Oregon, United States; 4 PSU School of Public Health, Portland, Oregon, United States; 5 Brown University School of Public Health, Providence, Rhode Island, United States

## Abstract

We identified a cohort of 410,413 Medicare beneficiaries residing in 10,623 large (25+bed) assisted living (AL) communities between 2007 and 2017. We conducted linear probability models with a difference-in-difference framework to examine the association between hospitalization and changes in regulations pertaining to staff training (model 1) and staffing levels (model 2), adjusting for time trends, resident characteristics, and state-license fixed effects. During this 11-year period, six states changed their staff training requirements and two states introduced/increased direct care staffing levels. A change in regulations related to staffing levels was associated with a reduction in the probability of hospitalization during the month of -0.0056 percentage points (95%CI=-0.008,-0.003). A change in regulations related to staff training was associated with a reduction in the probability of hospitalization during the month of -0.0035 percentage points (95%CI=-0.006,-0.002). The policy effects represent clinically important differences of approximately 21% in the mean monthly hospitalization rate. Part of a symposium sponsored by Assisted Living Interest Group.

